# Association of physical component score with high-risk lung nodules among Chinese Urban sanitation workers: a sex-specific analysis

**DOI:** 10.3389/fpubh.2026.1816641

**Published:** 2026-04-14

**Authors:** Liji Wu, Quhua Yin, Yufeng Yao, Siqin, Hong Zhang, Jiao Yang, Dongyi Xin, Ruifeng Jin, Feng Liu, Xinchen Zhao, Liwei Wang, Jian Du, Furong Wang

**Affiliations:** 1 Inner Mongolia Fourth Hospital & Inner Mongolia Chest Hospital, Hohhot, China; 2 Hunan Institute for Tuberculosis Control & Hunan Chest Hospital, Changsha, China; 3 Innovation Alliance on Tuberculosis Diagnosis and Treatment, Beijing, China; 4 International Mongolian Hospital of Inner Mongolia, Hohhot, China; 5 Inner Mongolia Autonomous Region Center for Disease Control and Prevention, Hohhot, China; 6 Beijing Chest Hospital, Capital Medical University, Beijing, China

**Keywords:** gender difference, Low-dose computed tomography, lung nodules, physical component summary, sanitation workers

## Abstract

**Background:**

Sanitation workers face chronic occupational exposure to ambient air pollution and traffic-related particulate matter; however, the prevalence of high-risk lung nodules in this vulnerable population remains unclear. Furthermore, the potential association between physical health-related quality of life (HRQoL) and nodule risk, along with its sex-specific patterns, has not been adequately investigated.

**Methods:**

This cross-sectional study included 1,018 outdoor sanitation workers in Hohhot, Inner Mongolia Autonomous Region, China. All participants underwent low-dose computed tomography (LDCT) screening and completed the SF-8 Health Survey. Lung nodules were assessed by two independent radiologists in a blinded manner. High-risk lung nodules (Lung-RADS Category 4) were confirmed by at least two senior specialists. Multivariate logistic regression and interaction analyses were employed to evaluate the association between Physical Component Summary (PCS) scores and high-risk lung nodules, adjusting for age, sex, smoking status, and socioeconomic factors.

**Results:**

A total of 16 participants (1.57%) were identified with high-risk lung nodules, of whom 9 (56.3%) were never-smoking females. The fully adjusted model included 994 participants. Multivariable logistic regression revealed an inverse association between PCS and high-risk lung nodules. Treated as a continuous variable, PCS showed a marginal inverse association after adjusting for sex, age, smoking, education, and residence (OR = 0.92, 95% CI: 0.84–1.00, *P* = 0.0505). When dichotomized at a cutoff of 50, a significantly decreased risk was observed in the PCS ≥ 50 group vs. the PCS < 50 group, which persisted after full adjustment (OR = 0.29, 95% CI: 0.10–0.83, *P* = 0.0211). Generalized additive models indicated a significant, nearly linear relationship (*P* = 0.027). Furthermore, subgroup analyses showed this protective effect was accentuated in females (OR = 0.87, 95% CI: 0.79–0.96, *P* = 0.0065) and highly educated individuals (OR = 0.81, 95% CI: 0.70–0.94, *P* = 0.0042), both yielding significant interactions (*P* for interaction = 0.0407 and 0.0319, respectively). Interactions for age, income, smoking, BMI, and residence were non-significant.

**Conclusion:**

A higher PCS is inversely associated with high-risk lung nodules, demonstrating a generally approximate linear relationship. This inverse association is more pronounced in females and individuals with higher educational levels, suggesting potential effect modification by sex and education.

## 1 Introduction

The increasing adoption of low-dose computed tomography (LDCT) has dramatically improved the detection of high-risk lung nodules. Given their malignant potential as precursors to lung cancer, these lesions represent a critical clinical and public health challenge. Emerging epidemiological evidence suggests that non-smoking-related factors—including environmental pollution (1, 2), occupational hazards (3), and genetic susceptibility—are increasingly recognized as key contributors to nodule formation, independent of traditional smoking risks. Notably, the incidence of high-risk lung nodules linked to occupational or environmental exposures is rising among non-smokers (4), with a disproportionate impact on women (5). From an occupational health standpoint, prolonged exposure to hazardous environments imposes a severe respiratory burden, highlighted by the cumulative pulmonary damage caused by occupational dust (6). Consequently, early identification and risk stratification remain a continuous clinical pursuit. While conventional risk assessment has heavily relied on imaging, clinical phenotypes, and molecular biomarkers—exemplified by prognostic models for lung malignancies (7, 8) and diagnostic tools for malignant pleural effusion (9)—a paradigm shift is underway. There is a growing emphasis on integrating functional metrics into chronic disease profiling. The recent development of machine-learning tools for managing sarcopenia (10) illustrates this shift, highlighting the prognostic utility of comprehensive health indicators in refining disease risk assessment.

However, current screening and risk assessment strategies for high-risk lung nodules predominantly target smoking populations and rely heavily on imaging, clinical characteristics, and molecular biomarkers. Consequently, specific occupational groups with elevated nodule risks driven by environmental or occupational exposures, such as sanitation workers, remain underexamined (11). In particular, there is a paucity of research investigating the association between comprehensive physical health status (e.g., body composition and functional capacity) and high-risk lung nodules, especially within occupationally exposed populations (12). Sanitation workers are chronically exposed to various airborne pollutants, including traffic exhaust, road dust, fine particulate matter (PM2.5), and ozone (O_3_) (13), which predisposes them to substantial respiratory health risks (14, 15). Nevertheless, systematic investigations into the prevalence characteristics of high-risk lung nodules and their correlation with systemic health in this specific demographic remain scarce.

The Physical Component Score (PCS), a comprehensive metric of overall health, has been robustly linked to the onset and progression of various diseases (16). It provides a direct and holistic evaluation of multidimensional physiological domains, encompassing muscle mass, pulmonary function, and metabolic status (17). PCS is significantly correlated with objective health markers (such as sarcopenia and reduced lung function), which are, in turn, established risk factors for the development and progression of lung nodules. This provides a theoretical foundation for systematically exploring the association between PCS and lung nodule risk across both general and high-risk screening populations. Despite this, epidemiological evidence remains sparse, with a notable absence of comprehensive analyses that integrate PCS assessment, nodule screening, and sex differences within specific occupational cohorts.

Therefore, this cross-sectional study was designed to systematically evaluate the prevalence of high-risk lung nodules among urban sanitation workers in China. Furthermore, we aimed to explore the association between PCS and the risk of high-risk lung nodules, with a particular focus on sex-specific differences. The findings are expected to provide empirical evidence for optimizing pulmonary health assessments, identifying risk factors, and formulating personalized health support strategies tailored to this occupational group.

## 2 Materials and methods

### 2.1 Study design and population

**Figure 1:**
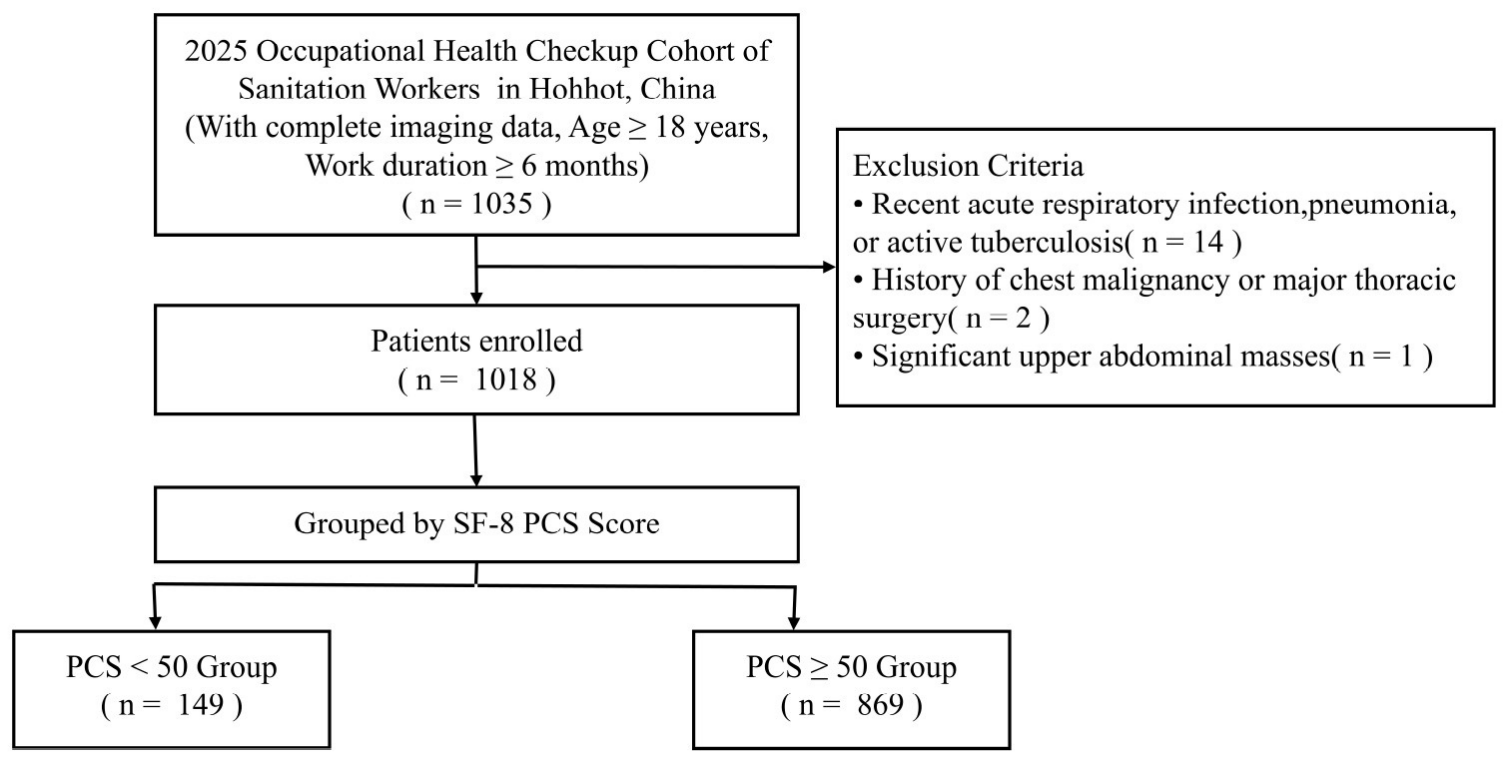
Flow chart of patient inclusion.

This cross-sectional study was conducted based on a cohort of in-service sanitation workers who participated in the 2025 annual occupational health examination in Hohhot, Inner Mongolia, China. Participants meeting the following criteria were enrolled: (1) continuous employment in the current position for at least 6 months; (2) age ≥18 years; and (3) voluntary participation in and completion of chest low-dose computed tomography (LDCT). The exclusion criteria included: (1) presence of acute respiratory infection symptoms within the past 2 weeks or radiographic evidence suggesting active pneumonia or pulmonary tuberculosis; (2) history of thoracic malignancy or major thoracic surgery; and (3) presence of severe upper abdominal space-occupying lesions. This study enrolled 1,018 eligible participants for the subsequent analysis (Figure 1).

### 2.2 Data collection and variable definitions

A structured occupational health survey was utilized to gather baseline information. We recorded participant demographics (age, sex) and physical parameters (height, weight) to determine Body Mass Index (BMI). Additionally, lifestyle behaviors were assessed, including current smoking (defined as smoking at least one cigarette daily for a minimum of 6 months). We utilized the SF-8 instrument to compute Physical and Mental Component Summary scores (PCS and MCS). Socioeconomic stratification involved four variables: education (High school or above vs. Middle school or below), annual income (≥30,000 RMB vs. < 30,000 RMB), marital status (married vs. unmarried/other), and residential area (urban vs. rural).

### 2.3 Imaging assessment and outcome definition

All participants underwent standard chest LDCT screening. high-risk lung nodules were defined in accordance with Lung-RADS v2022 (18) and C-Lung-RADS (19), nodules categorized as Lung-RADS 4A, 4B, and 4X were considered high-risk. To ensure diagnostic accuracy and objectivity, the following assessment workflow was established: All LDCT images were independently reviewed in a double-blind manner by two radiologists with over 5 years of experience, following current guidelines. Any cases initially identified as high-risk lung nodules or presenting with controversial findings were submitted to a Multidisciplinary Team (MDT) for final clinical adjudication. The MDT comprised at least two senior specialists from the departments of Radiology, Respiration, Infectious Diseases, and Thoracic Surgery. The final consensus reached by the MDT served as the definitive basis for the “high-risk nodule” classification.

### 2.4 Statistical analysis

Statistical analyses were conducted using R software (The R Foundation) and EmpowerStats (X&Y Solutions, Inc.). Participants were stratified into two groups for comparison using a PCS score of 50 as the cutoff point (≥50 vs. < 50). The rationale for this 50-point threshold derives from the norm-based scoring of the SF-8, in which scores below the population average of 50 are indicative of physical functional impairment. We employed Multiple Imputation by Chained Equations (MICE) to generate five imputed datasets to handle these missing confounders, and strictly applied Rubin's rules to pool the analytical results. Continuous variables were expressed as means with standard deviations (SD) or medians with interquartile ranges (IQR), and categorical variables were presented as frequencies and percentages. Differences between the two groups were evaluated using independent-samples *t*-tests or Kruskal-Wallis rank-sum tests for continuous variables, depending on data distribution. For categorical variables, Pearson's chi-square tests were employed, while Fisher's exact test was utilized when the theoretical frequency in any cell was less than 10. To explore potential non-linear associations between PCS scores and the risk of high-risk lung nodules, we used a generalized additive model (GAM) withsmooth curve fitting.

To evaluate the association between PCS scores (treated as both continuous and categorical variables) and outcome variables, we performed unadjusted and adjusted logistic regression analyses. The associations were quantified using odds ratios (ORs) and 95% confidence intervals (CIs).Three models were constructed: a crude model without adjustment for confounders; Model I adjusted for sex, age; and Model II further adjusted for location, smoking status, and educational level. Furthermore, to explore potential effect modifications and the robustness of the findings, stratified analyses were performed across various subgroups: sex, age (< 60 vs. ≥60 years), BMI (< 24 vs. ≥24 kg/m^2^), education level, income, smoking status, and residential location. Multivariable logistic regression models were utilized to calculate the odds ratios (ORs) and 95% confidence intervals (CIs) for each stratum, adjusting for potential confounders (including smoking, sex, age, education, and location, except for the stratifying variable itself in each model). Furthermore, interaction tests were conducted by incorporating cross-product terms (PCS × stratifying variable) into the models to evaluate whether the association between PCS and the outcome significantly differed across subgroups. All statistical tests were two-sided, and a *P*-value < 0.05 was considered statistically significant.

## 3 Results

### 3.1 Patient characteristics

The baseline demographic and clinical characteristics of the study population, categorized by PCS scores, are presented in Table 1. Of the total participants, 149 had a PCS score < 50, while 869 had a PCS score ≥50. Most baseline variables, including age, BMI, sex, and income, were comparable between the two groups (all *P* > 0.05). Regarding residential location, the lower PCS group had a significantly larger proportion of urban residents (85.23% vs. 76.92%, *P* = 0.024). Furthermore, although not reaching statistical significance, there was a trend suggesting that the lower PCS group tended to have lower educational attainment (*P* = 0.077) and a lower proportion of smokers (***P*** = 0.055) compared to their counterparts (Table 1).

**Table 1:** Baseline characteristics of participants by physical component summary (PCS) scores.

Variable	PCS < 50 (*N* = 149)	PCS ≥50 (*N* = 869)	*P*-value
AGE (Mean ± SD)	59.03 ± 7.67	59.26 ± 7.82	0.741
BMI (Mean ± SD)	25.72 ± 3.93	25.39 ± 3.67	0.320
PCS (Mean ± SD)	45.70 ± 3.60	56.83 ± 3.34	< 0.001
MCS (Mean ± SD)	49.38 ± 5.33	51.80 ± 3.11	< 0.001
**SEX**, ***n*** **(%)**			0.485
Male	71 (47.65%)	441 (50.75%)	
Female	78 (52.35%)	428 (49.25%)	
**LOC**, ***n*** **(%)**			0.024
Urban	127 (85.23%)	650 (76.92%)	
Rural	22 (14.77%)	195 (23.08%)	
**EDU**, ***n*** **(%)**			0.077
High (High school or above)	49 (32.89%)	219 (25.92%)	
15.6-7.4,-14.3242ptLow (Middle school or below)	100 (67.11%)	626 (74.08%)	
**SMOKE**, ***n*** **(%)**			0.055
Non-smoker	124 (83.22%)	661 (76.06%)	
Smoker	25 (16.78%)	208 (23.94%)	
**INCOME**, ***n*** **(%)**			0.919
High (≥30,000 RMB)	60 (40.27%)	344 (40.71%)	
Low (< 30,000 RMB)	89 (59.73%)	501 (59.29%)	

BMI, Body Mass Index; PCS, Physical Component Summary; MCS, Mental Component Summary.

Values are presented as Mean ± SD for continuous variables or *N* (%) for categorical variables. *P* values were calculated using independent-samples *t*-tests for continuous variables and chi-square tests for categorical variables.

### 3.2 Associations between PCS scores and the risk of high-risk lung nodules

When analyzed as a continuous variable, a higher PCS score was associated with a decreased risk of high-risk lung nodules in the crude model (OR = 0.92, 95% CI: 0.85–0.99, *P* = 0.0266). This inverse association remained significant after adjusting for age and sex in Model I (*P* = 0.0333). In the fully adjusted model (Model II), which further controlled for location, smoking status, and educational level, the association became marginally significant while maintaining a consistent protective trend (OR = 0.92, 95% CI: 0.84–1.00, *P* = 0.0505).

Furthermore, when PCS was evaluated as a categorical variable, higher PCS scores (≥50) consistently demonstrated a robust protective effect across all models. Compared to participants with a PCS score < 50, those with a PCS score ≥50 had a significantly lower risk of high-risk lung nodules. Specifically, in the fully adjusted Model II, having a PCS score ≥50 was associated with a 71% reduction in the odds of high-risk lung nodules (OR = 0.29, 95% CI: 0.10–0.83, *P* = 0.0211) (Table 2).

**Table 2:** Associations of continuous and categorical PCS Scores with the risk of high-risk lung nodules.

Exposure	Crude model	Model I	Model II
PCS (continuous)			
OR (95% CI)	0.92 (0.85, 0.99)	0.92 (0.85, 0.99)	0.92 (0.84, 1.00)
***P*** value	0.0266	0.0333	0.0505
PCS categorical			
< 50 (Reference)	1.0	1.0	1.0
≥50			
OR (95% CI)	**0.28 (0.10, 0.78)**	**0.28 (0.10, 0.78)**	**0.29 (0.10, 0.83)**
***P*** value	**0.0145**	**0.0152**	**0.0211**

OR, odds ratio; CI, confidence interval; PCS, physical component summary.

Model I: Adjusted for sex, age. Model II: Adjusted for sex, age, location, smoking status, and educational level. Bold values show PCS ≥50 significantly lowers high-risk lung nodule risk, persisting even after adjusting for confounding factors.

**Figure 2:**
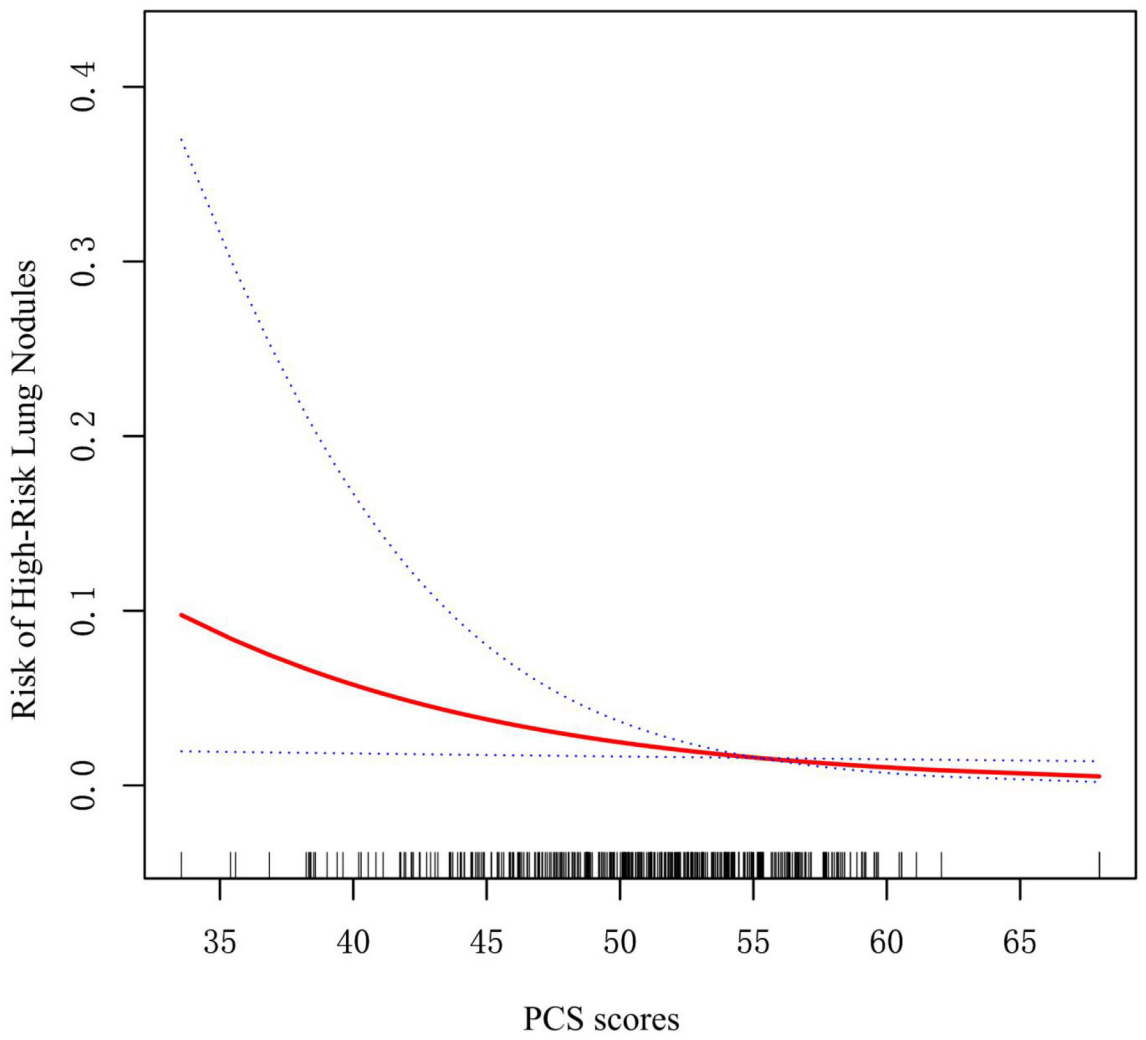
Dose-response relationship between continuous Physical Component Summary (PCS) scores and the risk of high-risk lung nodules. A generalized additive model (GAM) was used to evaluate this association. The solid line represents the estimated odds ratio (OR), and the dashed lines represents the 95% confidence intervals (CIs).

To further characterize the dose-response association between continuous PCS scores and the risk of high-risk lung nodules, generalized additive models (GAMs) were employed. As depicted in Figure 2, the unadjusted analysis revealed a significant, nearly linear inverse relationship (P = 0.027) (Figure 2).

### 3.3 Subgroup analyses of the association between PCS scores and the risk of high-risk lung nodules

Significant effect modifications by sex and educational level were observed (*P* for interaction = 0.0407 and 0.0319, respectively). The protective association of higher PCS scores against high-risk lung nodules was specifically prominent in females (OR = 0.87, 95% CI: 0.79–0.96, *P* = 0.0065), whereas no significant association was found in males (OR = 1.11, 95% CI: 0.86–1.43, *P* = 0.4163). Similarly, regarding educational attainment, a significantly decreased risk was evident only among participants with a high educational level (high school or above; OR = 0.81, 95% CI: 0.70–0.94, *P* = 0.0042), but not in those with a lower educational level (*P* = 0.8273).

Furthermore, while the interaction tests did not reach statistical significance for age, income, and smoking status (all *P* for interaction >0.05), stratified analyses revealed that higher continuous PCS scores were significantly associated with a reduced risk of high-risk lung nodules within specific subgroups. These protective associations were particularly evident among participants aged < 60 years (OR = 0.87, 95% CI: 0.76–0.99, *P* = 0.0327), those with higher income (≥30,000 RMB; OR = 0.84, 95% CI: 0.72–0.98, *P* = 0.0274), and non-smokers (OR = 0.91, 95% CI: 0.83–1.00, *P* = 0.0475). Conversely, no significant associations were observed across the strata of BMI and residential location (Table 3).

**Table 3:** Subgroup analyses of the association between continuous PCS and the risk of high-risk lung nodules.

Subgroups	*N*	OR (95% CI)	*P* value	*P* for interaction
Sex				
Male	493	1.11 (0.86, 1.43)	0.4163	**0.0407** ^*^
Female	501	0.87 (0.79, 0.96)	**0.0065** ^*^	
Education				
High (High school or above)	268	0.81 (0.70, 0.94)	**0.0042** ^*^	**0.0319** ^*^
Low (Middle school or below)	726	0.99 (0.88, 1.11)	0.8273	
Age				
< 60 years	425	0.87 (0.76, 0.99)	**0.0327** ^*^	0.2630
≥60 years	569	0.96 (0.85, 1.08)	0.4781	
Income				
High (≥30,000 RMB)	404	0.84 (0.72, 0.98)	**0.0274** ^*^	0.1863
Low (< 30,000 RMB)	590	0.95 (0.86, 1.06)	0.3492	
Smoking status				
Non-smokers	767	0.91 (0.83, 1.00)	**0.0475** ^*^	0.6511
Smokers	227	0.96 (0.78, 1.19)	0.7008	
BMI				
< 24 kg/m^2^	398	0.91 (0.81, 1.02)	0.1079	0.8148
> = 24 kg/m^2^	596	0.93 (0.82, 1.05)	0.2245	
Location				
Urban	777	0.93 (0.84, 1.01)	0.0944	0.6861
Rural	217	0.88 (0.72, 1.09)	0.2359	

OR, Odds Ratio; CI, Confidence Interval; PCS, Physical Component Summary; BMI, Body Mass Index. All models were adjusted for sex, age, location, smoking status, and educational level, except for the stratifying variable itself. The *P* for interaction indicates the significance of the interaction term between PCS and the respective stratifying variable. The asterisk (^*^) denotes statistical significance (*P* < 0.05) for either the subgroup association (*P* value) or the interaction effect (*P* for interaction). Bold values show PCS ≥50 significantly lowers high-risk lung nodule risk, persisting even after adjusting for confounding factors.

## 4 Discussion

In this study of 1,018 Chinese urban sanitation workers, the detection rate of high-risk lung nodules was 1.57%, highlighting severe respiratory risks within this occupational group. Previous literature has largely focused on broad symptom surveys. For example, a 2024 systematic review by Tolera et al. reported a 32.56% global pooled prevalence of occupational respiratory diseases among sanitation workers, with a disproportionate burden in low-income countries (20). Similarly, global analyses show respiratory issues are their leading health threat, comprising 52% of occupational hazard studies (21). Our findings support these epidemiological trends and, importantly, translate these generalized risks into specific pathological evidence of lung nodules via LDCT screening.

Chronic exposure to road dust, traffic pollution (e.g., PM2.5), and potential domestic cooking fumes (2, 20, 22) likely exacerbates the inherent health vulnerability of sanitation workers. Zhang et al. noted that pollutants like PM2.5 promote cellular malignancy via oxidative stress, DNA damage, and inflammatory responses (e.g., Wnt/β-catenin pathway activation). Coupled with the established carcinogenicity of heavy metals and PAHs in particulate matter (22, 23), these mechanisms may accelerate the malignant progression of high-risk lung nodules into lung cancer. Supporting these pathways, Pei et al. found a significant positive association between long-term PM2.5 and O3 exposure and lung nodule prevalence (PM2.5 OR = 1.06; O_3_ OR = 1.49) in Shijiazhuang, China. This association was partially mediated by inflammatory markers like platelets (PLT) and the neutrophil-to-lymphocyte ratio (NLR), suggesting that occupational air pollution provokes sustained systemic inflammation, thereby initiating or accelerating lung nodule growth (2).

Surprisingly, over half of the cases (56%) occurred in non-smoking women. This aligns with emerging global epidemiological trends showing a rising incidence of lung adenocarcinoma among never-smoking women, particularly in Asian populations. Based on the Chinese National Lung Cancer Screening Program (*N* = 796,283), Wu et al. found that for adenocarcinoma, known risk factors (e.g., air pollution) exerted a 7% higher pathogenic effect in females than males (24). This suggests women possess higher biological susceptibility to non-tobacco environmental carcinogens, predisposing them to localized pulmonary oxidative stress, high-risk nodule formation, and eventual cancer progression. Furthermore, a meta-analysis by Triphuridet et al. highlighted that LDCT lung cancer detection rates in Asian female never-smokers are comparable to those in male smokers (RR = 1.22) (25), typically diagnosed at earlier stages (26, 27). This confirms that Asian female never-smokers constitute an independent high-risk group, warranting targeted attention in lung nodule screening and follow-up.

Our core analysis demonstrated a robust, near-linear negative association between physical health status (assessed via PCS) and high-risk nodule risk. Notably, this protective effect peaked at PCS ≥50, reducing the risk by 71% (OR = 0.29), with significant effect modification by sex and education. This likely reflects a bidirectional vicious cycle of “physical impairment and psychological stress” (20, 25, 28). On one hand, lung nodules pose an uncertain health threat, triggering substantial psychological burden. Yuan et al. and Wu et al. highlighted that incidental nodule detection frequently causes moderate distress, anxiety, depression, and sleep disturbances (affecting 44.9% of patients); such chronic psychosomatic stress may promote nodule progression via immunosuppressive mechanisms (23, 29, 30). The ImaLife study similarly linked low-HRQoL factors (e.g., physical inactivity) with larger solid nodules (31). On the other hand, occupational exposure itself is an independent risk factor for anxiety (OR = 4.15) (28). Sanitation workers thus endure a “double blow” of physical environmental harm and psychological pressure, driving down HRQoL and elevating nodule risk. Furthermore, female patients exhibit significantly higher rates of nodule-related anxiety (21.57%) and depression (18.05%) than males (32, 33). Coupled with women's aforementioned heightened biological susceptibility to environmental carcinogens, a higher PCS—indicative of superior physical functioning—may provide a stronger “physiological buffer” in females against exposure-induced oxidative stress and airway inflammation. This elucidates the more pronounced PCS–nodule inverse association observed in the female subgroup (OR = 0.87). Concurrently, the amplified benefit among highly educated workers (*P*_interaction_= 0.0319) underscores the indispensable role of health literacy. Higher education typically translates into a better understanding of occupational hazards, increased compliance with personal protective equipment, and healthier lifestyle choices (34), generating an optimal synergy with a robust physiological baseline.

These findings may have practical implications for optimizing occupational health strategies for sanitation workers. First, given the high detection rates and specific risks among Asian female never-smokers, occupational LDCT screening criteria must expand beyond traditional male smokers to cover this vulnerable demographic, echoing calls to revise current guidelines (25). Second, for individuals with detected nodules, mere radiological follow-up is insufficient. As Tao et al. demonstrated, cognitive behavioral group therapy (CBGT) significantly ameliorates distress (35). Therefore, occupational health programs should integrate psychological interventions—particularly for female workers with low PCS—to decisively disrupt the vicious cycle of “anxiety-immunosuppression-progression” (30). Finally, regarding source control, governments must enforce occupational safety standards and upgrade protective equipment to fundamentally mitigate respiratory exposure hazards (20).

Several limitations warrant consideration. First, the cross-sectional design precludes establishing causality between HRQoL and high-risk lung nodules; reverse causality—where declining HRQoL results from, rather than causes, nodule formation—cannot be ruled out. Second, the sample is restricted to Chinese urban sanitation workers. Their unique demographics and specific environmental exposures (e.g., regional sandstorms) limit the generalizability of our findings to other geographic areas or occupational cohorts. Third, relying on Lung-RADS Category 4 to define high-risk lung nodules, while highly indicative of malignancy, is not equivalent to pathological diagnosis; actual malignant progression requires longitudinal validation. Finally, the absence of detailed occupational variables (e.g., tenure, protective equipment compliance) and objective environmental monitoring data renders our mechanistic explanations regarding occupational exposure somewhat speculative.

Moving forward, research should establish multi-center, prospective longitudinal cohorts to dynamically track the natural history (or dynamic evolution) of lung nodules in sanitation workers and elucidate causal links with HRQoL. In parallel, integrating environmental exposomics is essential to precisely quantify occupational exposure to agents like PM2.5 and polycyclic aromatic hydrocarbons (PAHs). Moreover, exploring metabolomic biomarkers could identify early warning indicators for lung cancer risk. Finally, given the sex disparities in screening outcomes and overdiagnosis risks, future intervention models should prioritize sex-specific strategies. Developing refined, life-course occupational health protection strategies specifically for female sanitation workers is warranted.

## 5 Conclusion

In conclusion, this study demonstrates a robust, near-linear negative association between physical health status (PCS) and the risk of high-risk lung nodules among sanitation workers. Notably, maintaining a PCS ≥50 confers a substantial protective effect (a 71% risk reduction), which is particularly pronounced in females and highly educated workers. Furthermore, our findings highlight Asian female never-smokers as an unexpected yet highly vulnerable demographic within this occupational cohort. These insights suggest the value of optimizing current occupational health strategies. Tailored approaches—such as considering the inclusion of non-smoking female workers in LDCT screening programs, providing psychological support for individuals with lower HRQoL, and continuously enhancing workplace source-control measures—may offer practical pathways to better protect the comprehensive wellbeing of this essential workforce.

## Data Availability

The raw data supporting the conclusions of this article will be made available by the authors, without undue reservation.
